# Understanding reactions to an internet-delivered health-care intervention: accommodating user preferences for information provision

**DOI:** 10.1186/1472-6947-10-52

**Published:** 2010-09-17

**Authors:** Lucy Yardley, Leanne G Morrison, Panayiota Andreou, Judith Joseph, Paul Little

**Affiliations:** 1 School of Psychology, University of Southampton, Southampton, UK; 2 School of Medicine, University of Southampton, Southampton, UK

## Abstract

**Background:**

It is recognised as good practice to use qualitative methods to elicit users' views of internet-delivered health-care interventions during their development. This paper seeks to illustrate the advantages of combining usability testing with 'theoretical modelling', i.e. analyses that relate the findings of qualitative studies during intervention development to social science theory, in order to gain deeper insights into the reasons and context for how people respond to the intervention. This paper illustrates how usability testing may be enriched by theoretical modelling by means of two qualitative studies of users' views of the delivery of information in an internet-delivered intervention to help users decide whether they needed to seek medical care for their cold or flu symptoms.

**Methods:**

In Study 1, 21 participants recruited from a city in southern England were asked to 'think aloud' while viewing draft web-pages presented in paper format. In Study 2, views of our prototype website were elicited, again using think aloud methods, in a sample of 26 participants purposively sampled for diversity in education levels. Both data-sets were analysed by thematic analysis.

**Results:**

Study 1 revealed that although the information provided by the draft web-pages had many of the intended empowering benefits, users often felt overwhelmed by the quantity of information. Relating these findings to theory and research on factors influencing preferences for information-seeking we hypothesised that to meet the needs of different users (especially those with lower literacy levels) our website should be designed to provide only essential personalised advice, but with options to access further information. Study 2 showed that our website design did prove accessible to users with different literacy levels. However, some users seemed to want still greater control over how information was accessed.

**Conclusions:**

Educational level need not be an insuperable barrier to appreciating web-based access to detailed health-related information, provided that users feel they can quickly gain access to the specific information they seek.

## Background

Qualitative research is recognised as vital in the development of all e-health interventions, and may be used for initial elicitation of stakeholder needs and desires, assessment of lay users' and experts' views regarding the content and format of interventions during development, and evaluation of user experiences of the completed intervention [1]. In particular, methods of 'usability testing' which originated in the field of human-computer interaction are widely considered an essential part of intervention development. The aim of usability testing is to identify and eliminate barriers to easy, safe and efficient use by members of the target population, and to establish user acceptability and satisfaction with the intervention [2-4]. A common qualitative technique for usability testing is the 'think aloud' method, which involves asking users to vocalise their reactions and thinking processes while, or immediately after, they use online resources [5,6]. This method may be supplemented by ethnographic observation, observation and timing of task completion, semi-structured interviews, probing questions, or brief surveys of users' views [2,4,7-9].

In-depth usability testing can yield crucial insights into the way in which the design of interventions needs to be adapted to the knowledge and capabilities of users. For example, Rotondi and colleagues (2007) found that users with cognitive impairment could navigate websites more easily if they deviated from standard best practice website design, for example by providing longer but more explicit labels for links and avoiding complex site structures. Similarly, observation of older people using a diabetes self-management system at home [8] revealed fundamental usability problems associated with anxiety about using the equipment (e.g. sitting too far from the computer screen, being reluctant to touch buttons) and confusion about the meaning of blood pressure readings. Usability testing has been employed effectively to develop a web-based decision aid that was accessible to older African-American men [10]. However, usability analysis has historically focused primarily on the cognitive and perceptual-motor processes (e.g. comprehension, skills, actions) that are required to accomplish goals [3]. Consequently, while user views of other aspects of interventions may be briefly reported, the traditional approach to usability testing is not ideally suited to exploring psychosocial factors influencing the experience of interventions. Moreover, it is often restricted to a pragmatic attempt to identify any common and serious problems with the usability of each iteration of the intervention, based on just a few users [11].

While studies of the usability of individual websites have been complemented by in-depth qualitative studies of how people view and use the internet and interventions [12-16], such studies have not generally been linked to usability testing. However, many developers of internet interventions are now interpreting usability more broadly, inviting general impressions and views of the content, tone and elements of the interventions [7,17-23]. These developmental studies have tended to retain an applied focus, and the findings they have generated have therefore been presented within a mainly practical and often website-specific framework. Nevertheless, the issues raised by users' reactions to internet-delivered interventions have wider implications for our understanding of the role and limitations of digital technology in healthcare, and the social, cultural and emotional factors influencing how interventions are experienced. By linking analysis of user views more explicitly to relevant social science theory and research, it should be possible to exploit the process of usability testing in order to gain greater insight into the psychosocial factors influencing users' reactions, and also to generalise more widely from studies of specific interventions [24].

This kind of analysis is likely to be particularly relevant to the 'theoretical modelling' stage of developing complex interventions [25,26], which involves analysing the theoretical basis for processes of change in order to understand precisely how and why the intervention should influence behaviour. Theoretical modelling can include any qualitative or quantitative empirical work that draws on theory to analyse processes affecting the delivery and receipt of an intervention. In the studies reported here, we first used qualitative methods inductively, to identify potentially important processes without making a priori assumptions about what these might be. Since attitudes to information provision emerged as an important theme, we linked our findings to existing relevant theory and research in this field to generate hypotheses about how best to accommodate user information preferences. We then used further qualitative research to explore whether users purposively sampled from populations likely to have differing preferences for information provision would respond positively to the revised method of information provision suggested by the theoretical modelling process.

The two in-depth qualitative studies presented here were carried out to inform the development of our internet-delivered health-care intervention, which was designed to help users decide whether they needed to seek professional help for their cold and flu symptoms or whether they could self-care. In addition to this applied objective, we intended these cases to serve as a method of revealing and exploring issues with more general implications for the development of digitally-delivered health-care in the future. The first study elicited users' reactions to prototype pages presented in paper format. The immediate purpose was to obtain specific feedback about changes that needed to be made to our planned content and format. For the purpose of theoretical modelling, we also identified inductive themes with wider theoretical implications. Major emerging themes concerned dilemmas about how much information should be presented, how, and to whom. We therefore linked our findings to the literature on patient preferences for information-seeking by considering their application to the specific context of using the internet for self-assessment and self-care of acute illness. This theoretical modelling process was used to inform the design of a prototype website which we hypothesised should be able to accommodate the information preferences of diverse users. Users' views of this website were then elicited in the second study, which examined whether the website had successfully responded to limitations identified in the first study. In particular, our second qualitative study investigated the extent to which our website design met the information-seeking needs of users with different levels of education.

## Methods

### The internet-delivered intervention

#### Rationale

The website was designed to advise users whether there were medical indications that they should consult their doctor for their current symptoms of influenza and the common cold, and to provide information which could help them self-care if appropriate. We had two aims, which we believed could be compatible [27]. The first aim was to empower lay users by meeting their information needs more effectively, providing in-depth personalised information that was convenient to access at any time [28]. The second aim was to achieve the health service and medical objectives of promoting cost-effective and medically appropriate use of consultation time, and educating users about the benefits of avoiding taking antibiotics for minor infections. There is some evidence that both these aims can be partially met by providing information and advice in booklet form [29,30], by telephone [31] or over the internet [32,33]. However, the impact of previous interventions has generally been limited and there may be scope to improve the way in which information is provided. In particular, recent advances in technology make it possible to provide more detailed information which is automatically 'tailored' to the specific needs of the individual, in order to increase perceived personal relevance and avoid information overload [34].

### Theory-based content and format of the intervention

To ensure that the advice was safe and medically appropriate we drew on the latest evidence-based medicine (e.g. Cochrane systematic reviews, NICE guidelines) and the clinical expertise of members of the research team. The content of the information provided was also informed by psychological theory relevant to coping. We provided information on each aspect of symptoms identified by Leventhal's model [35] as important to self-regulation of illness, i.e. identity (characteristic symptomatology), cause, timeline, consequences, and possibilities for control or cure. Drawing on Bandura's social cognitive theory [36], we sought to increase confidence to self-care by providing in-depth information to enhance skills and capabilities for managing symptoms, and provided 'vicarious learning' information about the coping experiences of others who had used these self-care methods (e.g. in clinical trials).

For the format of the website, we used a hybrid design [37]. Users were first presented with pages introducing the aims of the site and presenting the credentials of the team that created it, particularly the last author (who is a GP and expert on management of colds and influenza). A set of questions and advice had been produced for each cold and flu symptom (i.e. cough, sore throat, runny nose etc.), and users were constrained to complete a set of questions for the symptom they sought advice for. In the first study users were then presented with advice about consultation and relevant information about the identity, cause, timeline, consequences, and control or cure of their particular symptoms, with options to request more detailed information. Users were then asked about their needs and preferences for symptom management (e.g. whether they wished to use natural remedies or over-the-counter medication), and received information tailored to these, again with options to request more details.

Linking the findings from the first study to existing theory and research (see Discussion section), we hypothesised that in order to meet the needs of people who differ in their desire for information it may be best to provide users with only essential personalised advice, delivered in accessible language and format [38], but with the choice to access more detailed information if wanted. The prototype website was therefore designed so that users received brief advice about whether they should see the doctor, and the likely cause of their symptoms, immediately after answering diagnostic questions about their symptoms. They could then go straight on to choose tailored information about their preferred form of self-management, or could also ask for more information about the identity, cause, timeline, consequences, and control or cure of their specific symptoms, and answers to common questions about colds and flu.

### Study 1

#### Participants and procedure

The participants were 21 adults (15 females and 6 males) aged between 18-62 years; twelve were students. Participants were recruited by advertisements inviting people with current or recent cold symptoms to view and comment on our draft advice. Initially, we used convenience sampling within the university, hence twelve of the participants were students. As the analysis progressed we employed theoretical sampling from comparison populations (including young non-students and older people) by placing advertisements in the local community and on web notice boards. The final number of participants was determined by the point at which no important new themes seemed to be emerging, i.e. the data had reached saturation.

The study was approved by the Research Ethics Committee of the School of Psychology, University of Southampton. Interviews were carried out by PA at the convenience of the participant, either in their home or at the university. Written informed consent was obtained, and all interviews were tape-recorded and fully transcribed.

Using the 'think aloud' approach, the planned web-pages were presented to participants by hand sequentially as they would appear online. The researcher introduced the task by explaining that participants should just say whatever they thought or felt about the draft pages as they went through them, and emphasised that critical feedback would be particularly helpful. Participants were asked to tick options for more information they wanted to see, to mimic clicking on optional pages on a website. Prompts to verbalise and follow-up questions were used to elicit elaboration. After the participants went through the materials, they were asked for general comments and demographic details were collected. The duration of the interviews was on average 50 minutes, and participants were compensated for their time with a £5 gift voucher.

### Analysis

Thematic analysis was used to summarise recurring patterns across participants [39,40]. We drew on several techniques from grounded theory [41,42], including open and in vivo coding, theoretical sampling, and constant comparison, but did not consider the data presented here suitable for developing a full grounded theory. All the transcripts were read thoroughly by LY and PA, and the data were then coded inductively by PA, grounding codes closely in the text, and documenting coding decisions in a coding manual. Codes that were relevant to the research topic (i.e. all comments on the website) and identified in more than one participant's transcript were clustered into themes. Codes and themes were discussed and agreed by both coders, after modification and recoding where this seemed appropriate. Finally, LY applied the method of constant comparison to examine the extent to which the themes reported here varied across participants, and contextual factors associated with such variation. The resulting interpretation was reviewed and agreed with all authors.

The analysis presented below focuses on five emerging themes which concerned positive and negative views of providing in-depth information. The positive themes were: 'the information is helpful, reassuring and trustworthy'; 'could identify with the information provided - matches personal experience' and 'the information is interesting or useful for future reference'. The negative themes were 'information provided is excessive, overwhelming' and 'excessive information impedes accessing advice quickly'. These themes were selected for the analysis reported here because we believed that these findings had wider theoretical relevance to the development of online interventions in general. The remaining themes were not reported here as they were not relevant to the topic of this paper, and had chiefly practical utility in terms of improving this particular website: these were related to specific page content or format; general website format (e.g. colour, navigation, terminology); whether the participant agreed with the advice given on whether to consult the doctor; reflection on the circumstances in which they would consult the doctor rather than (or in addition to) the website; and specific suggestions for altering, updating or expanding what the website provided [43].

### Study 2

#### Participants and procedure

The procedure for this think aloud study was very similar to Study 1. The interviews were carried out by LM and JJ using the interactive website, and lasted between 45 and 90 minutes. The study had approval from Research Ethics Committee of the School of Psychology, University of Southampton, participants gave written informed consent, and were given £10 compensation for their time.

Participants were purposively recruited to sample a wide range of educational levels using advertisements around the university and city centre, personal approaches to people visiting pharmacies in deprived areas, and snowballing. The resulting sample consisted of were 26 adults (14 female and 12 male) aged between 18-63 years; 22 were Caucasian, and internet use ranged from 0 to 63 hours a week. See Table 1 (and quotations) for details of highest qualifications and current occupation.

**Table 1:** Occupation and highest qualifications of participants in Study 2

	*n *(%)
Occupation	
Student: Undergraduate	2 (7.7)
Student: Postgraduate	7 (26.9)
Academia/Professional	3 (11.5)
Management	2 (7.7)
Administrative/Secretarial	2 (7.7)
Skilled Trade	4 (15.4)
Customer Service	2 (7.7)
Unemployed	4 (15.4)
Highest Qualification	
PhD	3 (11.5)
Masters Degree	3 (11.5)
Bachelor Degree	6 (23.1)
Advanced academic school qualification (A level)	4 (15.4)
Basic academic school qualification (GCSE, O level)	3 (11.5)
Vocational qualifications (various)	7 (26.9)

### Analysis

Inductive thematic analysis was employed, as in study 1. The interview transcripts were coded by both LM and JJ, so to ensure consistency of coding a detailed coding manual was developed by LM, in discussion with JJ and LY. LM and JJ applied the coding manual independently to the data, discussed all instances of disagreement and resolved them, resulting in further elaboration of the coding manual. In discussion with LY, the inductive codes were then grouped into themes.

As in Study 1, the analysis presented here focuses only on the two themes concerned with views of the quantity and perceived usefulness of the information provided. LY again undertook constant comparison to examine variations between occurrences of themes across different participants and contexts (e.g. the context of the web-pages comments referred to). This stage of the analysis included a specific examination of whether positive or negative views of the information were related to education, occupation or gender, and a search for 'disconfirming cases' or exceptions to dominant patterns of responses [44].

## Results

### Study 1

#### Positive responses to the information provided

The information provided in the draft web-pages was in general enthusiastically received, and all participants commented favourably on some aspect of the information. Detailed information about the causes, significance and natural history of symptoms was seen as likely to reduce anxiety and support informed decision-making regarding whether and when it would be advisable to consult the GP rather than self-care. Some participants felt that this might save them the inconvenience of an unnecessary visit to the doctor:

You are put at ease, because you know what it is and what is happening so -- if you don't know what's happening to you then it can be, make it seem a lot worse than actually is. (RW2, male, 18, student)

I think that's fine because it tells me the reason, it tells me when I, it tells me that I don't need to see the doctor, it tells me what I would look for. (JT1, female, 24, MSc student)

That's quite reassuring, because even though my symptoms didn't last for more than seven days, if they came back I wouldn't be so worried, perhaps I wouldn't go back to see the GP, knowing that it's sort of normal to reoccur. (SW, female, 19, student)

The level of detail allowed participants to corroborate the information by comparing explanations for symptoms and treatment rationales against their personal experience. Although getting information that was already familiar was not always welcomed, participants often responded positively when the information was consonant with past experience. This verified that the information the website provided was correct and trustworthy, but more importantly reassured them about the validity of their existing beliefs and their self-care strategies. In this respect, the information was also regarded as a useful resource for supporting future self-management:

I would read this and I would see many things that I do by myself and I would say, "Oh, I'm doing it good. Oh, I have done this, oh, nice. Oh, yeah, my mum told me to drink honey and lemon" and things like that so I guess people will feel identified with these things. (FV, female, 25, PhD student)

That's helpful, even though I already knew that already, but it's helpful because it tells you exactly what causes the problem, 'cause you know next time that happens you know, you can be more aware yourself and you can be, you know you go to the chemist and take lozenges, or to just take paracetamol to help that - yeah, it's really good. (TB, female, 19, student)

'You can check for further information if your symptoms change' [website text] -- that's good, because you feel that, it can be some kind of, you know ongoing support easily accessible, if you come to worry about changes. (CR, female, 60, non-graduate)

### Negative responses to the information provided

Despite the widespread appreciation of information described above, many participants felt overwhelmed by the information. Some rejected the quantity provided, feeling that it was tedious and unnecessary:

It's too much for a first page, I'm already bored [laughs] it's too complicated, I'm a simple person. (SA, male, 52, non-graduate)

It's helpful if you're reading things for the first time and you're finding things for the first time. In other ways is a bit boring, especially for some things that are pretty common sense and you don't learn something new. (CH, female, 30, graduate)

The quantity of information was seen by some as a barrier to accessing the key points of the advice:

I think maybe the text is a bit too long, I mean I'd rather just get to the point straight away, and say 'You don't need to see the doctor' on a bullet point, and then the available treatments on the next page, and then if further just click here. (JA, male, 24, non-graduate)

Nevertheless, many who found the information daunting suggested more accessible ways in which it could be presented:

I think if I would see a whole page of text I kind of think "Oh, where do I start?", suppose I start from the top I'd be looking for the bits that stick out in some kind of a box with a big font or something. (JT6, male, 38, PhD student)

I suppose just the longer paragraphs at the beginning, just kind of cutting them down a bit, maybe people won't read if they just see a big chunk of text. (SW, female, 19, student)

### Study 2

The way in which the website presented information appeared to succeed in the aim to be more accessible, as general impressions of the website advice were positive among men and women of widely differing educational and occupational status:

There was a lot of information available, which I found was very good information. (3B, female, pharmacist, PhD)

I am quite impressed about how informative it is. (2C, male, unemployed, vocational qualification - NVQ level 2)

My general feeling is, there's a lot of really useful information on here. (6A, female, student)

The information that was there, that was good information, so the content I suppose you could say I like, the information was good. (P12B, male, shop worker, GCSE)

I did like the site and I certainly will go to the site again. (P4C, female, home-maker, Portugese high school certificate)

The element of choice may have been important in this respect; having personally chosen to see information users were likely to be receptive to it. For example, after clicking on information about whether antibiotics might be helpful, a female Turkish PhD student (10B) commented that:

I think it's very useful information, maybe it's the most useful information for me in the website I mean. Because I mean, most of the people really think that antibiotics will help.

The part of the website that proved most popular was the advice on 'treatment options'. Again, users seemed to appreciate the opportunity to select from a wide range and quantity of advice in this section, and some actually had an appetite for more information:

It's good, it's got everything you would want to click on I think. It gives you three different options to help yourself. (1A, male, scaffolder, vocational qualification - City and Guilds)

I think they're really interesting 'cause they give you some information on things that your doctor wouldn't necessarily tell you because of maybe he wouldn't think of it or wouldn't feel it's important for you to know. (3A, female, student)

It's quite basic, but that will probably just come with development as you sort of add more and more information on the website, then it's gonna become more useful - it's quite basic advice so it wouldn't be worth using it more than a few times. (7B, female, student)

While almost all interviewees expressed some positive views of the website advice, some still felt that parts of the website should be more concise. These comments were most often elicited by the pages of diagnostic questions (which could be quite extensive), and sometimes the introduction pages that explained the concept of the website and how to use it. There was still no evidence at all of any differences due to socioeconomic status, nor gender. Moreover, opinions were mixed, with some interviewees welcoming the scope of the diagnostic questions, and others acknowledging their utility despite wishing they could be less extensive:

I am starting to answer the questions quickly and not really think about them so much because there are quite a few questions. (4B, male, PhD student)

There's quite a lot of questions in there but I understand you need to ask that for medical reasons, I don't mind asking answering [sighs] answering questions because obviously you don't want that advice to be wrong. (1A, male, scaffolder, vocational qualification - City and Guilds)

This is going very in depth which is a good thing. (4A, male, student)

[referring to introductory pages] I want to do it quickly but I can't, I have to read everything. (9B, male, Indian PhD student)

Although negative responses to the treatment options section were rare, one woman who had found the website daunting throughout even found selecting treatment advice stressful (although she actually did welcome some parts of the advice):

I am a bit worried if I tick those there is going to be a lot more to read

[Interviewer: How does that make you feel?]

A bit stressed, but, you can find out too much, can't you?

[after she had ticked an option]

Oh God!

[Interviewer: What are you thinking now?]

Too much! There is too much, too many questions, too many, too many things to think about. (1B, female, secretary, O level)

## Discussion

### Understanding our findings in the context of information-seeking research

Reactions to the draft web-pages in Study 1 suggested that the detailed information they provided did appear to offer users some of the empowering benefits we intended, by allowing them easy access to extensive medical knowledge [13,28]. Participants' comments suggested that this did increase their confidence in the advice provided, and in their ability to cope with their symptoms. However, there was also feedback that the quantity of information was often experienced as excessive and off-putting. This creates a dilemma for optimal self-care website design; how much information should be presented, to whom, and in what way?

Theory and research on information-seeking, online and offline, is clearly relevant to this issue. Preferences for information-seeking are known to be related to demographic and psychological factors. Information-seeking is associated with a desire to take an active part in health-related decisions and control of health problems, and is more common in women, younger people and those with more education and higher incomes [45-47]. Barriers to information-seeking include lack of confidence in the ability to understand and use the information correctly, and feeling that taking personal responsibility for digesting health-related information could be anxiety-provoking, harmful and inappropriate [14,47,48]. However, it would be simplistic to categorise people (especially on the basis of education or class) as liking or disliking information - as was evident from the mixture of positive and negative comments often made by the same participants in our interviews. Individual differences in preference for information form a continuum rather than a dichotomy, and vary depending on the context and the type of information concerned [45,46,49].

Linking the findings from our first study to this understanding of information-seeking, we came to the conclusion that one way to satisfy the needs of people with differing preferences for information might be to provide users of our website with just a brief, accessible summary of the most essential personalised advice [38], but with the option to access more detailed information if they wished to. It was encouraging to find in the second study that this website design did appear to deliver advice in a way that was positively received by users with widely differing levels of education.

### Implications for enhancing the accessibility of self-care websites

Health literacy can be viewed as an interaction between the capabilities of individuals and the characteristics of their communication environment [50]. Consequently, by improving the accessibility of health websites it should be possible to diminish the literacy problems that contribute to the 'digital divide' [28,47], which may be partly sustained by an information environment on the web that is currently best suited to those with higher levels of education. Research suggests that other sectors of the population that are relatively low internet users at present, such as older people and those with cognitive impairment, are open to the possibility of using the internet for self-care, and can successfully do so when provided with an appropriately designed web environment [9,51,52].

Website developers are already aware of the need to make websites accessible in terms of readability of content, visual appearance, navigability and other aspects of usability. Our study adds to this body of knowledge by exploring how the way in which the website is designed to present information may also affect accessibility. Theory and research on health information-seeking suggests that the outcomes of information-seeking are optimal when people are able to access precisely the type and amount of information they want [45]. We found that by offering users more options to choose what information was viewed we could make the website format more appealing to those who did not want to be overloaded while still satisfying those who valued in-depth information. Similarly, [38] found that the website they designed to be more accessible to those with low literacy levels was also preferred by those with high literacy levels. Although some features of websites that improve accessibility may be disliked by those with higher education levels [9,38], a consideration for those creating health-care websites is that people who are anxious, tired or in pain may also benefit from a more accessible website [17]. Nevertheless, further research is needed in order to understand better the characteristics that will maximise the appeal and utility of website interventions for different groups of users.

### Dilemmas posed by user preferences for information control

Whereas information that was not wanted was criticised as excessive, information that was selected or was viewed as personally relevant, new and useful was highly valued. Hence, rejection of information as too lengthy may not be an absolute judgement but may rather convey the message that the value of the information is perceived as insufficient to justify the effort of processing it. One way that technology can be used to overcome this problem is to 'tailor' the information to the needs of the user [34]. Our website tried to reduce the quantity of information users had to confront by tailoring it to their specific symptom profile. The aim was partly to ensure the advice was medically appropriate to their symptoms, but also to make it more concise and personally relevant. However, this entailed asking users a series of questions that were themselves viewed as excessive by those who were not convinced that the advice they would receive would be worth the time spent completing the questions - a problem that has been encountered by other health self-care websites [53].

A third motive for designing the website to use questions about symptoms to provide tailored advice was to virtually recreate the traditional consultation, in which the doctor asks the patient questions and then gives personalised advice based on expert knowledge. By doing so, we hoped to provide a model of internet-based care that would be more acceptable to those who avoided seeking health information on the internet because they disliked taking an active role in self-diagnosis and choosing self-care options [14,48]. Unfortunately, by making these initial questions compulsory we inadvertently enforced a traditional, passive relationship to the medical authority of the website which may have been experienced as disempowering, since it reduces active user control [24]. Those users who found the diagnostic questions unnecessary but valued the treatment advice may have been signalling a desire for further choice and control, in this case over whether they would be obliged to engage in the website-guided diagnostic process. Rejection of this process by some users seems entirely logical, since by consulting a website rather than a doctor they are independently deciding that they are unlikely to have a serious condition that urgently requires medical advice, and are hence already taking up an active decision-making role [49]. The implication is that internet-delivered self-care may need to extend users' choice (where medically safe and appropriate) to include whether they wish to receive tailored advice from an 'expert' system, or prefer to self-diagnose and self-select information about management of their condition. - although this can also pose problems [53]. However, explicitly assigning responsibility for diagnosis to the user entails a profound shift in the way in which medical advice is made available to lay people and poses difficult questions regarding the management of risk, since users who self-diagnose may select medically inappropriate symptom management strategies. Moreover, there is already evidence that taking key medical decisions (such as whether a condition is serious) is naturally often difficult and anxiety-provoking for people with limited medical knowledge [46,49,53].

## Conclusions

The 'theoretical modelling' approach we adopted can be considered a hybrid of usability testing and in-depth qualitative research, intended to enhance the generalisability and depth of insight that can be derived from analysing data from usability studies. The end result should be not only a better web-delivered intervention, but also insights that can contribute to more general principles for understanding and delivering self-care in the future. By linking to wider theory, we were able to generate hypotheses about likely influences on responses to the proposed website materials, such as individual differences and socio-demographic factors associated with preferences regarding active information-seeking. Then by modifying the website and re-examining responses to it in a large, educationally diverse sample we could begin to test the plausibility of these hypotheses and generate new ones. In this case, our findings suggest that educational level may not be an insuperable barrier to appreciating web-based access to in-depth self-care information, provided that users can feel they have sufficient choice and control and can quickly gain access to the specific information they value. These principles may well apply to all users, but the choices users make relating to what information they wish to view in which format are likely to vary, albeit not necessarily as a simple function of demographic characteristics.

## Competing interests

The authors declare that they have no competing interests.

## Authors' contributions

LY led and supervised both studies and drafted the manuscript. LM, PA and JJ carried out the data collection and initial analysis. PL co-supervised the studies and led the medical input into the design of the website. All authors contributed to and approved the final manuscript.

## Pre-publication history

The pre-publication history for this paper can be accessed here:

http://www.biomedcentral.com/1472-6947/10/52/prepub
